# Wearable Evidence Linking Dyskinesia Burden to Sleep Quality in Parkinson's Disease

**DOI:** 10.1002/mdc3.70887

**Published:** 2026-11-01

**Authors:** Viktoria Azoidou, Essa Bhadra, Ellen Camboe, Kamalesh C. Dey, Alexandra Zirra, Kira Rowsell, Corrine Quah, Caroline Budu, Thomas Boyle, David Gallagher, Jonathan P. Bestwick, Laura Pérez‐Carbonell, Alastair J. Noyce, Cristina Simonet

**Affiliations:** ^1^ Centre for Preventive Neurology, Wolfson Institute of Population Health Queen Mary University of London London UK; ^2^ Department of Neurology The Royal London Hospital, Barts Health London UK; ^3^ Neurology Department Homerton Healthcare NHS Foundation Trust London UK; ^4^ Older Person's Services & GIM Special Interest in Parkinson'’ Disease Royal London Hospital London UK; ^5^ Sleep Disorders Centre Guy's and St Thomas’ NHS Foundation Trust London UK; ^6^ Unidad de Trastornos del Movimiento, Servicio de Neurología, Instituto de Biomedicina de Sevilla Hospital Universitario Virgen del Rocío/CSIC/Universidad de Sevilla Sevilla Spain

**Keywords:** dyskinesia, Parkinson's disease, sleep quality, wearable devices

## Abstract

**Background:**

Sleep disturbances affect 60–80% of people with Parkinson's disease (PD) and worsen quality of life. Dyskinesia is a common complication of dopaminergic therapy, yet its link to sleep quality is poorly understood.

**Objectives:**

To examine whether clinician‐rated and objectively measured dyskinesia are associated with sleep quality in PD.

**Methods:**

We studied 47 people with PD (median age 68 years; 44.7% women; median disease duration 5 years). Sleep was measured with Pittsburgh Sleep Quality Index (PSQI). Dyskinesia was rated clinically (MDS‐UPDRS items 4.1, 4.2) and objectively over 7 days using Parkinson's KinetiGraph (median dyskinesia score; DKS). Regression models adjusted for age, sex, motor severity, levodopa dose, and disease duration.

**Results:**

All dyskinesia measures predicted poorer sleep unadjusted. After adjusting for levodopa dose, only DKS remained significant (*B* = 0.18, *P* = 0.047). Adding disease duration attenuated this (*P* = 0.051).

**Conclusions:**

Wearable‐derived dyskinesia, but not clinician‐rated dyskinesia, independently predicted poorer sleep, supporting wearable monitoring in PD.

Sleep disturbance is among the most prevalent non‐motor features of Parkinson's disease (PD), affecting up to 80% of patients within 5 years of diagnosis.^1^, ^2^ A pooled estimate placed poor sleep quality at 58% across 9382 patients.^3^ Sleep disturbances in PD arise from neurodegeneration of sleep–wake regulatory circuits, dopaminergic medication effects, and nocturnal motor symptoms, and independently predict worse health‐related quality of life.^4^, ^5^

Levodopa‐induced dyskinesia (LID) is a motor complication of treated PD. In the COPPADIS cohort of 672 patients, LID increased from 18.9% at baseline to 42.6% at 5 years,^6^ and population‐based data show that around 30% of levodopa‐treated patients develop dyskinesia at a median of 4 years from levodopa initiation.^7^ Dyskinesia development is increasingly understood as a function of disease duration rather than cumulative levodopa exposure.^8^, ^9^, ^10^, ^11^

Sleep and dyskinesia have been linked in both clinical and neurophysiological data. In a previous cross‐sectional study, poor nighttime sleep was independently associated with dyskinesia in 425 PD patients,^12^ and prospectively, a high PSQI score predicted incident dyskinesia at 36 months.^13^ Impaired sleep‐dependent synaptic downscaling, reflected in a blunted overnight slow‐wave activity decline, has been proposed as a possible shared mechanism.^14^, ^15^

However, prior studies linking sleep quality to dyskinesia have relied on clinician‐rated or patient‐reported assessments. MDS‐UPDRS items 4.1 and 4.2 combine history and observation to estimate whole‐day dyskinesia but depend on patient report and may underestimate motor burden.^16^, ^17^, ^18^ The Parkinson's KinetiGraph (PKG) is a validated wrist‐worn accelerometer that samples motor activity every 2 min over 7–10 continuous days. Its dyskinesia output has been validated against the Abnormal Involuntary Movement Scale.^18^

No prior study has compared continuous wearable‐derived and clinician‐rated dyskinesia as correlates of global subjective sleep quality within the same cohort. We therefore examined whether dyskinesia burden, assessed by both approaches, was associated with subjective sleep quality in people with PD.

## Methods

This cross‐sectional study included 47 patients with idiopathic PD recruited from specialist outpatient clinics at Barts Health NHS Trust and Homerton Healthcare NHS Foundation Trust. PD was diagnosed according to Movement Disorder Society clinical diagnostic criteria.^19^ All participants provided written informed consent, and the study was approved by the London‐Dulwich Research Ethics Committee (23/PR/1526).

Subjective sleep quality was assessed using the PSQI, a validated measure of global sleep quality over the preceding month.^20^ The PSQI total score was prespecified as the primary outcome. Motor complications were assessed using Part IV of the Movement Disorder Society‐Unified Parkinson's Disease Rating Scale (MDS‐UPDRS), with dyskinesia quantified using item 4.1 (time spent with dyskinesias) and item 4.2 (functional impact).^21^ Objective motor data were obtained from the PKG, worn continuously for 7 days. The median dyskinesia score (DKS) and Fluctuation Dyskinesia Score (FDS) were the wearable‐derived measures of dyskinesia burden.^18^ Median bradykinesia score (BKS), percent time immobile (PTI) and percent time with tremor (PTT) are reported descriptively.

Cognitive function was measured using the Montreal Cognitive Assessment (MoCA).^22^ Demographic and clinical variables also recorded were age, sex, self‐reported race/ethnicity,^23^ disease duration from formal diagnosis (years), Hoehn & Yahr stage,^24^ levodopa equivalent daily dose (LEDD), and MDS‐UPDRS Parts I‐III scores (covering non‐motor and motor experiences of daily living, and motor examination, respectively).^21^ MDS‐UPDRS Part III was assessed in the ON medication state.

Analyses were performed in IBM SPSS Statistics version 29. Continuous variables are presented as median (IQR), and categorical variables as frequencies and percentages. Because dyskinesia measures were non‐normally distributed, associations between PSQI total and each dyskinesia variable were examined individually using Spearman's rank correlations. Partial Spearman correlations were computed in three pre‐specified hierarchical adjustment models: Model A adjusted for age, sex, and MDS‐UPDRS Part III (motor examination), selected a priori as potential confounders. Model B added LEDD^25^ and Model C added disease duration. Models were ordered by temporal precedence. Multivariable linear regression was performed in parallel using the same Models A, B, and C, with PSQI total as the dependent variable. All tests were two‐sided (α = 0.05). No correction for multiple comparisons was applied given the exploratory design.

## Results

Forty‐seven patients with PD were included (Table 1). Median age was 68 years (IQR 62–76); female sex, 44.7%; median disease duration, 5 (2–8) years; median MDS‐UPDRS Part III, 45.0 (36.5–50.5), and median LEDD, 400 (300–500) mg/day. Dyskinesia severity was mild to moderate (median MDS‐UPDRS Part IV items 4.1 and 4.2 score 0 [0–2]; PKG DKS 1.40 [0.55–2.50]; FDS 8.10 [6.35–10.20]). Median PSQI was 10.0 (8.0–14.5), well above the validated cut‐off of five.^20^

**TABLE 1:** Demographic, clinical, and questionnaire characteristics

Characteristics		Participants (N = 47)
Demographic	Age (years)	68.00 (62.00–76.00)
	Sex	
	Male, n (%)	26 (55.30%)
	Female, n (%)	21 (44.70%)
	Ethnicity	
	White or White British (n, %)	29 (61.70%)
	Mixed or multiple ethnic group (n, %)	0 (0.00%)
	Asian or Asian British (n, %)	15 (31.90%)
	Black, African, Caribbean or Black British (n, %)	3 (6.40%)
	Disease duration from formal diagnosis (years)	5.00 (2.00–8.00)
Clinical	Hoehn & Yahr stage	3.00 (2.00–3.00)
	LEDD (mg/day)	400 (300–500)
	MDS‐UPDRS Part I	21.0 (11.5–25.0)
	MDS‐UPDRS Part II	20.0 (9.5–25.0)
	MDS‐UPDRS Part III	45.0 (36.5–50.5)
	MDS‐UPDRS Part IV	9.0 (6.0–14.0)
	MDS‐UPDRS Part IV (item 4.1)	0.00 (0.00–2.00)
	MDS‐UPDRS Part IV (item 4.2)	0.00 (0.00–2.00)
	MoCA	26.00 (24.00–27.00)
	PSQI	10.00 (8.00–14.50)
PKG‐derived measures	DK_50	1.40 (0.55–2.50)
	FDS	8.10 (6.35–10.20)
	BKS	28.10 (23.80–34.75)
	PTI (%)	9.50 (5.30–15.05)
	PTT (%)	1.90 (1.00–5.90)

*Note*: Data are presented as n (%) for categorical variables and median (interquartile range; IQR) for continuous variables.

Abbreviations: BKS, median bradykinesia score; DKS, median dyskinesia score; FDS, fluctuation dyskinesia score; Hoehn & Yahr stage, clinical measure of disease severity; LEDD, levodopa equivalent daily dose; MDS‐UPDRS Part III, motor examination; MDS‐UPDRS Part IV, motor complications with items 4.1 = time spent with dyskinesias and 4.2 = functional impact of dyskinesias; MDS‐UPDRS, Movement Disorder Society‐Unified Parkinson's Disease Rating Scale; MoCA, Montreal Cognitive Assessment; PKG, Parkinson's KinetiGraph; PSQI, Pittsburgh Sleep Quality Index; PTI, percent time immobile; PTT, percent time with tremor.

In unadjusted analyses, worse subjective sleep quality (higher PSQI) was associated with greater dyskinesia burden across clinical and wearable‐derived measures alike (SUPPLEMENTARY Table S1). PSQI correlated with MDS‐UPDRS Part IV item 4.1 (ρ = 0.386, *P* = 0.007), item 4.2 (ρ = 0.392, *P* = 0.006), and DKS (ρ = 0.487, *P* < 0.001). The association with FDS did not reach significance (ρ = 0.265, *P* = 0.072).

After adjusting for age, sex, and motor examination (MDS‐UPDRS Part III; Model A), the association between DKS and PSQI strengthened (ρ = 0.625, *P* < 0.001) (SUPPLEMENTARY Table S1). FDS reached borderline significance at this level of adjustment (ρ = 0.319, *P* = 0.035). In contrast, MDS‐UPDRS Part IV item 4.1 (ρ = 0.283, *P* = 0.063) and item 4.2 (ρ = 0.273, *P* = 0.073) were attenuated and no longer significant. After additional adjustment for LEDD (Model B), the DKS‐PSQI association remained strong (ρ = 0.577, *P* < 0.001), while the FDS association was attenuated (ρ = 0.257, *P* = 0.096). Clinician‐rated items remained non‐significant in Models A and B (item 4.1, *P* = 0.063–0.185; item 4.2, *P* = 0.073–0.200). After adjustment for disease duration (Model C), the DKS‐PSQI partial correlation remained significant (ρ = 0.580, *P* < 0.001), whereas item 4.1 (ρ = 0.202, *P* = 0.200), item 4.2 (ρ = 0.194, *P* = 0.219), and FDS (ρ = 0.251, *P* = 0.108) were not significant.

In multivariable linear regression adjusting for age, sex, MDS‐UPDRS Part III (Model A), and LEDD (Model B), DKS was the only independent predictor of PSQI (*B* = 0.18, 95% CI 0.003–0.357, *P* = 0.047). FDS (*B* = 0.40, 95% CI −0.03 to 0.83, *P* = 0.070), and MDS‐UPDRS Part IV item 4.1 (*P* = 0.187), and item 4.2 (*P* = 0.275) did not reach significance (Table 2). After adjustment for disease duration (Model C), DKS attenuated to borderline non‐significance (*B* = 0.18, 95% CI −0.001 to 0.356, *P* = 0.051), with FDS (*P* = 0.063), item 4.1 (*P* = 0.104) and item 4.2 (*P* = 0.142) non‐significant.

**TABLE 2:** Multivariable linear regression of PSQI on dyskinesia measures across covariate adjustment models

Model	Covariates	n	B	SE	β	95% CI	*P*
MDS‐UPDRS Part IV item 4.1							
Model A	Age + Sex + MDS‐UPDRS Part III	47	1.315	0.555	0.343	0.195 to 2.436	0.022
Model B	Model A + LEDD	47	0.834	0.621	0.218	−0.421 to 2.089	0.187
Model C	Model B + Disease duration	47	1.076	0.647	0.281	−0.231 to 2.384	0.104
MDS‐UPDRS part IV item 4.2							
Model A	Age + Sex + MDS‐UPDRS Part III	47	1.350	0.607	0.319	0.125 to 2.575	0.032
Model B	Model A + LEDD	47	0.769	0.696	0.182	−0.636 to 2.174	0.275
Model C	Model B + Disease duration	47	1.109	0.741	0.262	−0.388 to 2.606	0.142
PKG DKS							
Model A	Age + Sex + MDS‐UPDRS Part III	47	0.211	0.091	0.336	0.028 to 0.394	0.025
Model B	Model A + LEDD	47	0.180	0.088	0.286	0.003 to 0.357	0.047
Model C	Model B + Disease duration	47	0.177	0.088	0.282	−0.001 to 0.356	0.051
PKG FDS							
Model A	Age + Sex + MDS‐UPDRS Part III	47	0.522	0.211	0.380	0.096 to 0.949	0.018
Model B	Model A + LEDD	47	0.399	0.214	0.290	−0.033 to 0.831	0.070
Model C	Model B + Disease duration	47	0.410	0.215	0.298	−0.024 to 0.845	0.063

Abbreviations: B, unstandardized regression coefficient; CI, confidence interval; DKS, median dyskinesia score; FDS, fluctuation dyskinesia score; LEDD, levodopa equivalent daily dose; MDS‐UPDRS, Movement Disorder Society‐Unified Parkinson's Disease Rating Scale; PKG, Parkinson's KinetiGraph; PSQI, Pittsburgh Sleep Quality Index; SE, standard error; β, standardized regression coefficient.

## Discussion

Sleep disturbance and dyskinesia are common in PD and can each affect quality of life,^4^, ^6^ yet their association has been characterized almost exclusively through clinician‐rated or patient‐reported measures. We show that the choice of dyskinesia measurement method substantially changes the apparent dyskinesia‐sleep relationship in PD. To our knowledge, this is the first head‐to‐head comparison of continuous wearable and intermittent clinician‐rated dyskinesia as correlates of subjective sleep quality, and the first in a demographically diverse UK cohort.

Mao et al,^12^ in 425 patients, found that subjective sleep quality and sleep latency were the PSQI components most strongly associated with dyskinesia. Tang et al^13^ prospectively showed that high PSQI predicted incident LID at 36 months. This evidence together with our findings point towards a bidirectional relationship. Amato et al^14^ reported a reduced overnight decline in slow‐wave activity in 14 dyskinetic patients, interpreted as impaired synaptic downscaling,^11^ but this small cross‐sectional study could not determine whether it precedes LID. Conversely, dyskinesia may itself disturb sleep. Our cross‐sectional design cannot establish the direction of this relationship.

Measurement differences may explain the attenuation of clinician‐rated associations. MDS‐UPDRS items 4.1 and 4.2 reflect the clinician's judgment of the whole‐day situation^21^ but depend on patient report and use a coarse ordinal scale on which more than half of our participants scored zero. The PKG instead quantifies dyskinesia directly across several days.^18^ The strengthening of the DKS‐PSQI association after severity adjustment is consistent with negative confounding, whereby OFF‐state‐dominant patients with little dyskinesia obscured the dyskinesia‐specific signal in unadjusted analysis.

Further indirect observations are consistent with a link between sleep and LID. In patients followed before any LID emerged, the magnitude of the overnight decline in slow‐wave activity predicted time to dyskinesia onset,^26^ a pattern paralleled in the 6‐OHDA rat model.^27^ Once dyskinesia is established, raised morning cortical theta further characterizes the dyskinetic state.^28^ Because DKS summarizes dyskinesia across several days, it may capture a sustained burden relevant to the one‐month recall window of the PSQI.

That the FDS‐PSQI association weakened once LEDD was accounted for suggests the fluctuation component is partly carried by medication load rather than reflecting an independent neurobiological signal.^29^ The attenuation by disease duration is harder to interpret cleanly, and three readings are compatible with our data. It may be a common‐cause confounder, since both LID risk and sleep disturbance rise with disease progression,^4^, ^8^ so adjusting for it removes shared variance. Alternatively, it may sit closer to the mechanism itself, standing in for the cumulative corticostriatal remodeling that drives both phenomena in which case adjusting for it constitutes over‐adjustment.^30^ A third reading is more prosaic: with 47 patients and five covariates, we are at the edge of what the data can support, and the near‐identical point estimates before and after adjustment are consistent with loss of power rather than abolition of the effect.

In a UK PD service introducing routine PKG monitoring, dyskinesia was detected in patients who had not previously reported it.^31^ Our data support incorporating wearable dyskinesia assessment into the care of patients with PD and persistent poor sleep, particularly when clinic‐based dyskinesia screening is negative.

Several limitations apply. The cross‐sectional design precludes causal inference, and the modest sample restricted covariate modeling, contributing to the borderline result after disease‐duration adjustment. Disease duration was measured from formal diagnosis rather than symptom onset, which likely underestimates the true biological duration. Median LEDD appeared low relative to disease duration and motor severity. In the Parkinson's Progression Markers Initiative,^32^ the mean MDS‐UPDRS Part III score was 32.5 after 5 years of disease duration. Median BKS was 28.1, as shown in Table 1, which might indicate that some participants may have been undertreated. This may have limited the observed range of dyskinesia severity and consequently attenuated associations towards the null. PTI, a PKG‐derived surrogate measure of daytime sleep that has been validated against polysomnography,^33^ may complement the PSQI in future studies. Sleep was assessed only by self‐report. The global PSQI obscures component‐level patterns and cannot capture sleep architecture. Unmeasured confounders including depression, anxiety, nocturia, restless legs syndrome, and ethnicity‐related non‐motor burden,^34^ may all influence both dyskinesia and sleep and warrant adjustment in future work. Strengths include continuous wearable dyskinesia measurement, pre‐specified hierarchical adjustment, a demographically diverse cohort.

In conclusion, greater dyskinesia burden was associated with worse sleep, but only wearable‐derived DKS, not clinician‐rated dyskinesia, remained a significant predictor after adjustment. These findings support continuous wearable monitoring in patients with persistent poor sleep. Larger prospective studies are needed.

## Author Roles

(1) Research project: A. Conception, B. Organization, C. Execution; (2) Statistical analysis: A. Design, B. Execution, C. Review and critique; (3) Manuscript: A. Writing of the first draft, B. Review and critique.

V.A.: 1A, 1B, 1C, 2A, 2B, 3A

E.B.: 1C, 2C, 3B

E.C.: 1C, 2C, 3B

K.C.D.: 1C, 2C, 3B

A.Z.: 1C, 2C, 3B

K.R: 1C, 2C, 3B

C.Q.: 1C, 2C, 3B

C.B.: 1C, 2C, 3B

T.B.: 1C, 2C, 3B

D.G.: 1C, 2C, 3B

J.P.B.: 2A, 2B, 2C, 3B

L.P.C: 1B, 2C, 3B

A.J.N.: 1A, 1B, 2C, 3B

C.S.: 1A, 1B, 2C, 3B

## Disclosures

**Ethical Compliance Statement:** Ethical approval for this study was granted by the London‐Dulwich Research Ethics Committee (reference: 23/PR/1526). All procedures were conducted in accordance with the principles of the Declaration of Helsinki. Written informed consent was obtained from all participants prior to enrolment. We confirm that we have read the position of *Movement Disorders Clinical Practice* on issues involved in ethical publication and affirm that this work is consistent with those guidelines.

**Financial Disclosures and Conflicts of Interest:** This study was supported by UK Research and Innovation through a Knowledge Transfer Partnership (Innovate UK, 2021–2022, Round 4). All authors report no personal fees, equity holdings, or advisory compensation related to this work. AJN does not have conflict of interest or disclosures to report in relation to this manuscript but he reports in general, not related to this work, research grant support from Parkinson's UK, Barts Charity, Cure Parkinson's, the National Institute for Health and Care Research, Innovate UK, UKRI, HORIZON 2020, the Medical College of Saint Bartholomew's Hospital Trust, Alchemab, Aligning Science Across Parkinson's/Global Parkinson's Genetics Program (ASAP‐GP2), and the Michael J. Fox Foundation within the past 3 years. AJN has also received consultancy or personal fees from AbbVie, Bial, and Roche. LPC does not have conflict of interest or disclosures to report in relation to this manuscript but reports, unrelated to this work, research grant support from the Michael J. Fox Foundation, director and speaker fees for the RBD Symposium at Guy's and St Thomas’ NHS Foundation Trust, consultancy fees from Takeda and Idorsia within the past 3 years. VA hold two, unrelated to this work, grants from National Institute for Health and Care Research School for Primary Care. Author disclosures are available in the Supporting Information.

## Supporting information


**Supplementary Table S1.** Associations between sleep quality and dyskinesia measures across covariate adjustment models.

**Data S1.** Supporting Information.

## Data Availability

De‐identified participant data and supporting documents including study protocol, statistical analysis plan, data dictionary, and consent form will be made available after publication upon reasonable request to the corresponding author, subject to a data use agreement and applicable ethical constraints.
