# Supplementary material for: Wearable Evidence Linking Dyskinesia Burden to Sleep Quality in Parkinson's Disease
Source: Mov Disord Clin Pract. 2026 Nov 1:10.1002/mdc3.70887. Online ahead of print. doi: 10.1002/mdc3.70887 (PMC13724529; doi:10.1002/mdc3.70887)
Supplement: Supplementary file 2 [file MDC3-9999-0-s002.docx]

**SUPPLEMENTARY TABLE S1. Associations between sleep quality and dyskinesia measures across covariate adjustment models**

| **Model** | **Covariate set** | **n** | **Partial ρ** | **P-value** |
| --- | --- | --- | --- | --- |
| **MDS-UPDRS Part IV item 4.1** | | | | |
| Unadjusted | (no covariates) | 47 | 0.386 | 0.007 |
| Model A | Age + Sex + MDS-UPDRS Part III | 47 | 0.283 | 0.063 |
| Model B | Model A + LEDD | 47 | 0.206 | 0.185 |
| Model C | Model B + Disease duration | 47 | 0.202 | 0.200 |
| **MDS-UPDRS Part IV item 4.2** | | | | |
| Unadjusted | (no covariates) | 47 | 0.392 | 0.006 |
| Model A | Age + Sex + MDS-UPDRS Part III | 47 | 0.273 | 0.073 |
| Model B | Model A + LEDD | 47 | 0.199 | 0.200 |
| Model C | Model B + Disease duration | 47 | 0.194 | 0.219 |
| **PKG DKS** | | | | |
| Unadjusted | (no covariates) | 47 | 0.487 | <0.001 |
| Model A | Age + Sex+ MDS-UPDRS Part III | 47 | 0.625 | <0.001 |
| Model B | Model A + LEDD | 47 | 0.577 | <0.001 |
| Model C | Model B + Disease duration | 47 | 0.580 | <0.001 |
| **PKG FDS** | | | | |
| Unadjusted | (no covariates) | 47 | 0.265 | 0.072 |
| Model A | Age + Sex + MDS-UPDRS Part III | 47 | 0.319 | 0.035 |
| Model B | Model A + LEDD | 47 | 0.257 | 0.096 |
| Model C | Model B + Disease duration | 47 | 0.251 | 0.108 |

MDS-UPDRS, Movement Disorder Society-Unified Parkinson's Disease Rating Scale; PSQI, Pittsburgh Sleep Quality Index; PKG, Parkinson's KinetiGraph; DKS, median dyskinesia score; FDS, fluctuation dyskinesia score; LEDD, levodopa equivalent daily dose; ρ, Spearman's rank correlation coefficient.
